# MIRD Pamphlet No. 27: MIRDcell V3, a Revised Software Tool for Multicellular Dosimetry and Bioeffect Modeling

**DOI:** 10.2967/jnumed.121.263253

**Published:** 2022-09

**Authors:** Sumudu Katugampola, Jianchao Wang, Alex Rosen, Roger W. Howell

**Affiliations:** Division of Radiation Research, Department of Radiology, New Jersey Medical School, Rutgers University, Newark, New Jersey

**Keywords:** dosimetry, radionuclide, multicellular cluster, cell survival, nonuniform activity distribution

## Abstract

Radiopharmaceutical therapy is growing rapidly. However, yet to be addressed is the implementation of methods to plan treatments for circulating tumor cells, disseminated tumor cells, and micrometastases. Given the capacity of radiopharmaceuticals to specifically target and kill single cells and multicellular clusters, a quality not available in chemotherapy and external-beam radiation therapy, it is important to develop dosimetry and bioeffect modeling tools that can inform radiopharmaceutical design and predict their effect on microscopic disease. This pamphlet describes a new version of MIRDcell, a software tool that was initially released by the MIRD committee several years ago. **Methods:** Version 3 (V3) of MIRDcell uses a combination of analytic and Monte Carlo methods to conduct dosimetry and bioeffect modeling for radiolabeled cells within planar colonies and multicellular clusters. A worked example is provided to assist users to learn old and new features of MIRDcell and test its capacity to recapitulate published responses of tumor cell spheroids to radiopharmaceutical treatments. Prominent capabilities of the new version include radially dependent activity distributions, user-imported activity distributions, cold regions within the cluster, complex bioeffect modeling that accounts for radiation type and subcellular distribution, and a rich table of output data for subsequent analysis. **Results:** MIRDcell V3 effectively reproduces experimental responses of multicellular spheroids to uniform and nonuniform distributions of therapeutic radiopharmaceuticals. **Conclusion:** MIRDcell is a versatile software tool that can be used for educational purposes and design of radiopharmaceutical therapies.

## Visual Abstract

**Figure:**
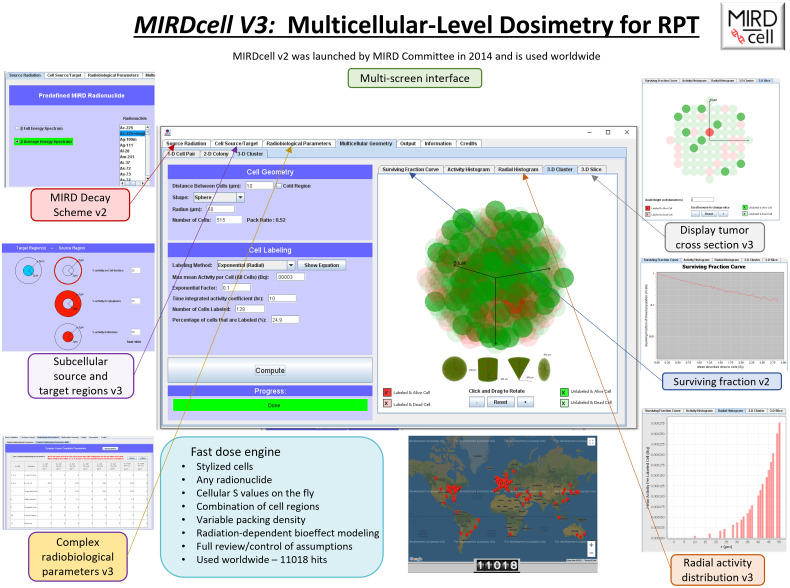

The widespread use of ^223^Ra-dichloride (Xofigo; Bayer) and ^177^Lu-DOTATATE (Lutathera; Advanced Accelerator Applications) has rejuvenated radiopharmaceutical therapy (RPT) of cancer. RPT delivers radioactive drugs to the primary tumor, metastases, disseminated tumor cells, and circulating tumor cells. Different classes of radionuclides are used for therapy, including α-, β-, and Auger electron emitters (*1*). The different ranges of these radiations in tissue, and their differences in relative biological effectiveness, contribute to the complexity of predicting therapeutic efficacy and normal-tissue toxicity (*1*). However, like external-beam radiation therapy, the future of RPT will depend in part on our capacity to plan treatments that maximize therapeutic effect while minimizing adverse effects on normal tissues. Key to the long-term success of RPT is to implement strategies that overcome limitations of the intrinsic nonuniform uptake of radiopharmaceuticals by cancer cells, which can impact our capacity to sterilize tumors, metastases, disseminated tumor cells, and circulating tumor cells.

Although treatment of primary tumors, distant metastases visible by external imaging, and microscopic metastases in locoregional lymph nodes can be addressed with external beams of radiation, most micrometastases, disseminated tumor cells, and circulating tumor cells cannot. There are commercial tools, based on external imaging, to assist with calculating absorbed dose to macroscopic disease in the context of both external-beam radiation therapy and RPT. The resulting absorbed doses have been used to predict response of tumor and normal tissues. However, there is a dearth of readily available tools that can be used to optimize and plan RPT of microscopic disease.

In 2014, the MIRD committee released version 2.0.15 of MIRDcell, a Java applet, to address the need for software tools for dosimetry and bioeffect modeling of microscopic disease treated with RPT. The software interface and its capabilities were described in MIRD pamphlet no. 25 (*2*). That version, and a later version, 2.0.16, ran on a web browser until 2017, when web browser support for Java applets was discontinued because of security concerns. Version 2.1 (V2.1), a Java application, was released in 2017 to eliminate the web browser requirement and permit the program to run as an application on the user computer.

This new version of our MIRDcell software application, version 3.10 (V3), was created in collaboration with the MIRD committee. MIRDcell V3 can run on all operating systems supporting Java. The software can model radiation absorbed dose and cell survival responses in single cells, cell pairs, and 2-dimensional (2-D) and 3-dimensional (3-D) cell populations. 2-D cell populations are constrained to lie on a plane (e.g., monolayer cell cultures), whereas 3-D populations can be organized within a variety of geometries. The organization of the tabs and the options within each tab, as well as other important details regarding the version history, are provided in detail in the downloadable user manual. The nomenclature used is consistent with dosimetric terminology published in MIRD pamphlet no. 21 (*3*). The app and user manual can be downloaded via https://mirdsoft.org or directly at https://mirdcell.njms.rutgers.edu/. The primary purpose of this present MIRD pamphlet is to describe some of the changes in interactive features, new activity distributions, and new bioeffect models that have been added to the software. More importantly, this pamphlet provides several examples of how to use these new features.

## MATERIALS AND METHODS

### Preamble

The distribution of radioactivity within small tissue elements can have a profound effect on the absorbed dose distribution and, correspondingly, the response of the tissue. Aside from other factors, the absorbed dose distribution and biologic response are strongly dependent on the type, yield, and energy of the radiations emitted by the radionuclide and its subcellular distribution. Most notable are radionuclides that decay by electron capture or internal conversion (e.g., ^111^In, ^123^I, ^125^I), which are followed by the emission of a shower of low-energy Auger electrons. Auger electrons deposit their energy over subcellular dimensions; therefore, these radionuclides invariably produce nonuniform absorbed-dose distributions at all spatial levels (*4**,**5*). Similarly, the short range of α-particles in biologic tissues (40–100 μm) also leads to nonuniform dose distributions from radionuclides such as ^223^Ra, ^225^Ac, and other α-particle emitters of potential use for RPT (*5*–*10*). Medium- and high-energy β-particle emitters such as ^177^Lu and ^90^Y have a greater degree of cross-irradiation because their mean range in tissue is at least several hundred microns. However, the nonuniform distribution of these radionuclides invariably leads to nonuniform dose distributions as well (*11*–*15*).

Although the distributions of absorbed dose that arise from nonuniform distributions of radioactivity are important, an additional factor that determines biologic response is whether a given absorbed dose arises from radioactive decays within a cell itself (self-dose) or from decays in surrounding cells or other parts of the body (cross-dose). The response of a cell to self-dose from a radiopharmaceutical can be different from its response to cross-dose from the same radiopharmaceutical. This difference is most notable for Auger electron emitters, for which the relative biological effectiveness for the self-dose can be an order of magnitude greater than the relative biological effectiveness for cross-dose (*16*). This observation has also been seen for DNA-incorporated β-particle emitters, for which the self-dose from ^131^I was 3 times more lethal than the cross-dose (*17*).

There is a growing body of experimental data on the biologic effects of nonuniform distributions of radioactivity at the multicellular level (*17*–*23*). These findings can have significant consequences for therapeutic uses of these and other radionuclides. MIRDcell V3 provides new tools that can be used to assist in understanding the dependence of radiopharmaceutical efficacy on numerous factors, such as radiation type and energy; distribution at the subcellular, cellular, and multicellular levels; and spatial arrangement of the cells within the multicellular structure (2-D plane [e.g., colony], 3-D cluster, and packing density). These capabilities and new bioeffect modeling features, which are expanded on through examples below, can be helpful in designing RPT strategies.

### “Source Radiation” Tab

The “Source Radiation” tab allows the user to select the radioactivity in the source cells (i.e., cells labeled with radioactivity). Three choices are available: predefined MIRD radionuclide, monoenergetic particle emitter, and user-defined radionuclide. User-defined radionuclides include decay chains for ^211^At, ^213^Bi, ^223^Ra, and ^225^Ac. Details on the differences between these options can be found in the user manual.

### “Cell Source/Target” Tab

As described in detail by Goddu et al. (*24**,**25*), cells are modeled as 2 concentric spheres with radii corresponding to those for the nucleus and cell, respectively. The cells are modeled as liquid water of unit density. The eligible source regions are cell, cell nucleus, cytoplasm, and cell surface. MIRDcell V3 newly permits the user to distribute the activity among cell nucleus, cytoplasm, and cell surface. The eligible target regions for which the radiation absorbed dose is calculated and used for bioeffect modeling are cell, cell nucleus, and cytoplasm. The addition of cytoplasm as a target is new to MIRDcell V3. No limit has been set on the maximum cell radius; however, extensive testing has been conducted only up to 10 μm. Although the algorithms should be adequate for calculating absorbed doses to larger spheres, caution should be exercised when interpreting results for cell radii larger than 10 μm. To facilitate this option, MIRDcell V3 now allows entering the radii in the text box. Users should be mindful that photons are ignored in this and earlier versions of MIRDcell; photon contributions to the absorbed dose can become significant for large sphere sizes.

### “Radiobiologic Parameters” Tab

MIRDcell enables the user to model the surviving fraction (SF) of cells in a specified cell population based on the calculated absorbed doses to the individual cells. Two options are available in V3 for calculating the probability that a given cell survives: simple radiobiologic parameters and complex radiobiologic parameters.

#### Simple Radiobiologic Parameters

As in MIRDcell V2.1 (*2*), a modified linear-quadratic (LQ) model is used to calculate the probability *P*(*r_k_*) that the *k_th_* cell survives a radiation absorbed dose to a region within, *r_k_* (*26**,**27*):

$$P(r_{k})=e^{-{\text{α}}_{\text{self}}D_{\text{self}}-{\text{β}}_{\text{self}}D_{\text{self}}^{2}}\times e^{-{\text{α}}_{\text{cross}}D_{\text{cross}}-{\text{β}}_{\text{cross}}D_{\text{cross}}^{2}},$$ Eq. 1

where α_self_ and β_self_ characterize the response of the cell to self-dose (*D*_self_), α_cross_ and β_cross_ characterize the cellular response to cross-dose (*D*_cross_), and the effect of self- and cross-dose are independent (*5**,**17**,**28*). The distinction between self- and cross-dose is often required for Auger electron emitters (*18**,**29*) and is sometimes required for β-particle emitters when they are DNA-incorporated (*17*). The determination of whether a given cell survives (alive) or not (dead) is determined by a Monte Carlo method by which the surviving probability, calculated using Equation 1, is compared with a randomly generated number.

#### Complex Radiobiologic Parameters (New)

A new feature of V3 is the capacity to specify LQ parameters not only for self-dose and cross-dose but also independently for each type of radiation (e.g., α, β, and Auger) and for each target region (cell [*C*], cell nucleus [*N*], and cytoplasm [*Cy*]). A modified LQ model is again implemented in V3.10. For example, when the cell nucleus is the target region and the source radiation type is designated by ICODE, the probability that the *k*^th^ cell survives the insult is given by Equation 2:

$$\begin{array}{l} P_{\text{ICODE}}\left(N_{k}\right)\\ =e^{-{\text{α}}_{\text{ICODE}}^{\text{self}}\left(N_{k}\leftarrow N_{k}\right)D_{\text{ICODE}}^{\text{self}}\left(N_{k}\leftarrow N_{k}\right)-{\text{β}}_{\text{ICODE}}^{\text{self}}\left(N_{k}\leftarrow N_{k}\right){\left[D_{\text{ICODE}}^{\text{self}}\left(N_{k}\leftarrow N_{k}\right)\right]}^{2}}\\ \times \;e^{-{\text{α}}_{\text{ICODE}}^{\text{self}}\left(N_{k}\leftarrow Cy_{k}\right)D_{\text{ICODE}}^{\text{self}}\left(N_{k}\leftarrow Cy_{k}\right)-{\text{β}}_{\text{ICODE}}^{\text{self}}\left(N_{k}\leftarrow Cy_{k}\right){\left[D_{\text{ICODE}}^{\text{self}}\left(N_{k}\leftarrow Cy_{k}\right)\right]}^{2}}\\ \times \;e^{-{\text{α}}_{\text{ICODE}}^{\text{self}}\left(N_{k}\leftarrow CS_{k}\right)D_{\text{ICODE}}^{\text{self}}\left(N_{k}\leftarrow CS_{k}\right)-{\text{β}}_{\text{ICODE}}^{\text{self}}\left(N_{k}\leftarrow CS_{k}\right){\left[D_{\text{ICODE}}^{\text{self}}\left(N_{k}\leftarrow CS_{k}\right)\right]}^{2}}\\ \times \;e^{-{\text{α}}_{\text{ICODE}}^{\text{cross}}\left({\text{N}}_{k}\leftarrow N_{j}\right)D_{\text{ICODE}}^{\text{cross}}\left(N_{k}\leftarrow N_{\text{numcell}}\right)-{\text{β}}_{\text{ICODE}}^{\text{cross}}\left(N_{k}\leftarrow N_{j}\right){\left[D_{\text{ICODE}}^{\text{cross}}\left(N_{k}\leftarrow N_{\text{numcell}}\right)\right]}^{2}}\\ \times \;e^{-{\text{α}}_{\text{ICODE}}^{\text{cross}}\left(N_{k}\leftarrow Cy_{j}\right)D_{\text{ICODE}}^{\text{cross}}\left(N_{k}\leftarrow Cy_{\text{numcell}}\right)-{\text{β}}_{\text{ICODE}}^{\text{cross}}\left(N_{k}\leftarrow Cy_{j}\right){\left[D_{\text{ICODE}}^{\text{cross}}\left(N_{k}\leftarrow Cy_{\text{numcell}}\right)\right]}^{2}}\\ \times \;e^{-{\text{α}}_{\text{ICODE}}^{\text{cross}}\left(N_{k}\leftarrow CS_{j}\right)D_{\text{ICODE}}^{\text{cross}}(N_{k}\leftarrow CS_{\text{numcell}})-{\text{β}}_{\text{ICODE}}^{\text{cross}}\left(N_{k}\leftarrow CS_{j}\right){\left[D_{\text{ICODE}}^{\text{cross}}(N_{k}\leftarrow CS_{\text{numcell}})\right]}^{2}}, \end{array}$$ Eq. 2

where *j* denotes another cell, numcell implies that the cross-dose can arise from all cells within the cluster, and

$$D_{\text{ICODE}}^{\text{self}}(N_{k}\leftarrow N_{k})=f_{N}\tilde{A}(C_{k})\;S_{\text{ICODE}}^{\text{self}}(N_{k}\leftarrow N_{k}).$$ Eq. 3

The ICODEs for the different radiation types are as defined in the *MIRD: Radionuclide Data and Decay Schemes* monograph (*30*). Here, $f_{N}$ is the fraction of cell activity in the nucleus, $\tilde{A}(C_{k})$ is the time-integrated activity in the source region $N_{k}$, and $S_{\text{ICODE}}^{\text{self}}\left(N_{k}\leftarrow N_{k}\right)\;$ is the self–S coefficient corresponding to the absorbed dose per decay from $N_{k}\leftarrow N_{k}$ and is given by Equation 4:

$$S_{\text{ICODE}}^{\text{self}}(N_{k}\leftarrow N_{k})=\overset{\text{irad}N}{\underset{\text{irad}=1}{\sum }}\frac{\Delta_{\text{ICODE,irad}}\phi_{\text{ICODE,irad}}(N_{k}\leftarrow N_{k})}{m(N_{k})},$$ Eq. 4

where the sum runs through all irad*N* radiations of type ICODE, $\Delta_{\text{ICODE,irad}}$ is the mean energy emitted per nuclear transition of the irad^th^ radiation of type ICODE, and $\phi_{\text{ICODE,irad}}\left(N_{k}\leftarrow N_{k}\right)$ is the fraction of energy emitted from the source region $N_{k}$ that is absorbed in the target region $N_{k}$ of the irad^th^ radiation of type ICODE. The terms corresponding to the self-dose from other cell compartments of the same cell (cytoplasm, cell surface) can be written similarly, as can the terms corresponding to the cross-doses from other cells. Finally, the overall probability of the *k*^th^ cell surviving, after the effects of all radiation types on the *k*^th^ cell nucleus *N_k_,* is written as follows:

$$P(N_{k})=\overset{\text{number}\;\text{of}\;\text{ICODEs}}{\underset{\text{ICODE}=1}{\prod }}P_{\text{ICODE}}(N_{k}).$$ Eq. 5

Here, MIRDcell adopts an independent interaction model in which the effect of each radiation type is considered independently of the other. As in prior versions of MIRDcell, the determination of whether a given cell survives is determined by a Monte Carlo method in which the probability of survival, calculated using Equation 5, is compared with a random number (0≤x≤1). The user manual provides details.

A complete set of equations for all possible scenarios of source and target regions is provided in the user manual. Default values are arbitrary, and the user is cautioned to enter values that are relevant to the application. The user is provided with the option of importing a desired set of LQ parameters and saving a set of custom parameters used in the model.

### “Multicellular Geometry” Tab

#### Cluster Geometry

As in MIRDcell V2.1, MIRDcell V3 has 3 basic geometric configurations of spherical cells: 1-dimensional (1-D), 2-D, and 3-D. These are summarized here, and details are provided in the user manual.

The 1-D option is presented in the “1-D Cell Pair” tab and is used to calculate the self- and cross-doses for a pair of cells. The user can set the distance between the centers of 2 cells. The self-dose and cross-dose S coefficients (formerly S values) are calculated using analytic methods based on range–energy relationships for electrons (*31**,**32*) and α-particles (*33*) as described in the supplemental materials (available at http://jnm.snmjournals.org).

The 2-D option is used to create a cell population that resides on a plane (i.e., colony). The cell-packing density can be specified by changing the distance between the cells, and the shape (circle, rectangle, ellipse) and dimensions of the colony can be set.

The 3-D option is attained by extending the planar cell configuration to a 3-D cluster. The shape of the cluster is selectable as a sphere, ellipsoid, rod, or cone cell-packing density, and the dimensions of the cluster are specified by the user. The cluster is assembled in a 3-D Cartesian coordinate system in a close-packed cubic lattice.

#### Cell Labeling

The distribution of activity among the labeled cell population is set by the user in the “2-D Cluster” and “3-D Cluster” tabs. As in MIRDcell V2.1, both the 2-D and the 3-D configurations offer several random distributions by which the activity is distributed among the labeled cells according to a uniform, normal, or lognormal distribution. Labeled cells are selected randomly, and each cell is randomly assigned an initial activity according to the user-selected distribution. A uniform activity distribution among the labeled cells implies that each labeled cell has the same initial activity *A* in its source region. In the normal distribution, the initial activity per cell is distributed according to the probability density function:

$$f\left(A\right)=\;\frac{1}{A\text{σ}\sqrt{2\text{π}}}e^{\frac{-{\left(A-\langle A\rangle \right)}^{2}}{2{\text{σ}}^{2}}},$$ Eq. 6

where 〈*A*〉 is the mean initial activity per cell and σ is the SD of the mean. In the case of the lognormal distribution, the activity per cell is distributed according to the probability density function:

$$f\left(A\right)=\;\frac{1}{A\text{σ}\sqrt{2\text{π}}}e^{\frac{-{\left(\mathrm{lnA}-\left(ln\langle A\rangle -{\text{σ}}^{2}/2\right)\right)}^{2}}{2{\text{σ}}^{2}}},\;A>0\;,$$ Eq. 7

where σ is the lognormal shape-parameter. The functional forms of the 3 distributions are best viewed in the “Activity Histogram” tab.

New functionality in MIRDcell V3 includes both built-in and user-provided radial activity distributions. The built-in radial distributions are linear, exponential, polynomial, and 4-parameter lognormal distributions. Polynomial distributions up to the 10^th^ degree are possible by setting the parameters accordingly. In all the radial activity distributions, a radius of 0 µm corresponds to the center of the cell cluster. The user-defined activity distribution feature is available only for the spherical cluster geometry. Furthermore, for the ellipsoid cluster geometry, only the standard normal, lognormal, and uniform activity distributions are available as cell-labeling methods. Details on each distribution are provided in the user manual.

MIRDcell V2.1 assumed that the radiopharmaceutical penetrates all the way into the cell cluster. MIRDcell V3 now provides the option of creating a cold region at the center of the cluster and specifying the depth (in μm) to which the drug penetrates the cluster from its outer surface. This situation is common for clusters with radii of more than 50 μm. The cold region at the center of the cluster will contain unlabeled cells. The various activity distributions described in the previous paragraph can be assigned to the cluster’s outer region, which has the labeled cells. The complex algebraic algorithms that are used to label cells according to the drug penetration depth are provided for different geometries in the user manual.

#### Visualization of Radial Distributions (New)

MIRDcell V3 now has tools to visualize the radial distributions of mean activity per labeled cell, mean self-dose to labeled cells, mean cross-dose to labeled cells, mean cross-dose to unlabeled cells, mean decays per labeled cell, and mean dose to all cells. This feature can be accessed from the “Radial Histogram” tab, which is available only for 3-D cluster geometries. It is an important tool for checking that the specified activity distribution meets the user’s expectations.

#### Visualization of Tomographic Sections (New)

**FIGURE 1.**
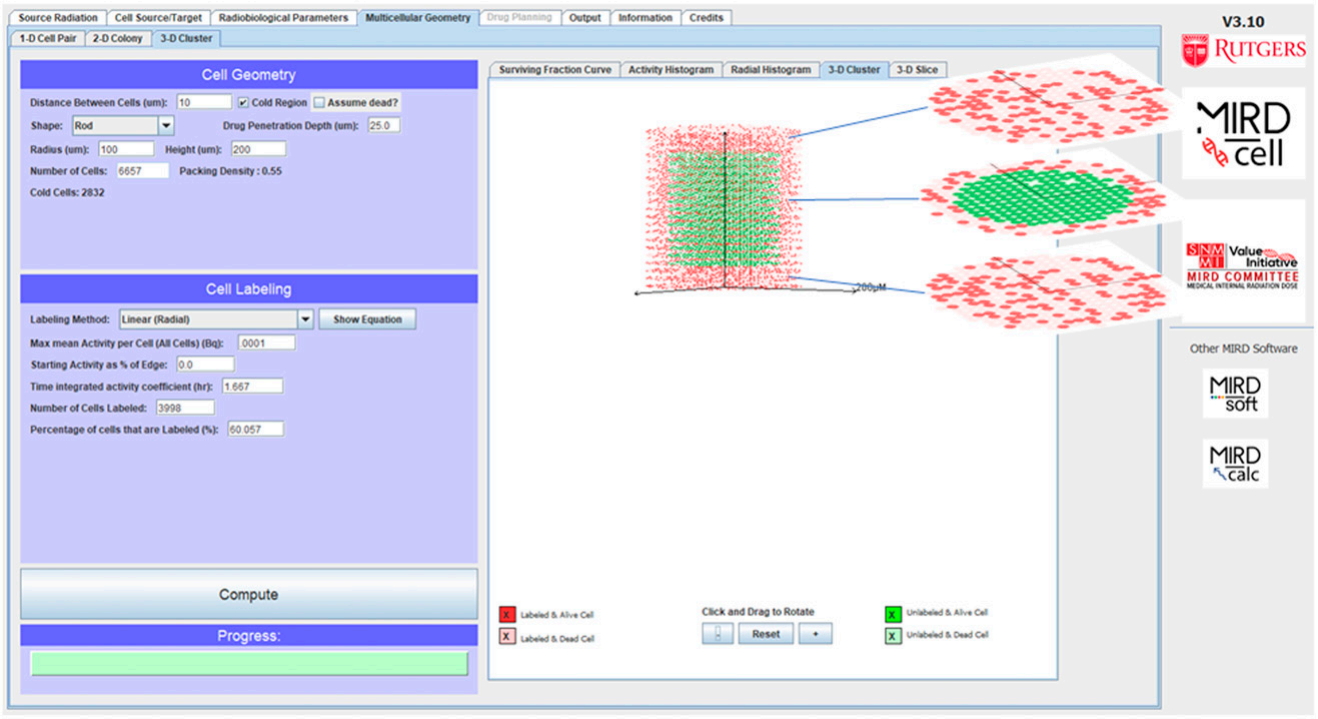
View of 3 tomographic sections of rod-shaped cluster of cells with cold region in interior. Red cells are labeled with radionuclide. Green cells are unlabeled. Opaque cells are alive, and translucent cells are dead. Blue lines point to tomographic section of corresponding cell layer.

Visual representations of the 3-D cell cluster with color-coded labeled/unlabeled and alive/dead cells is accessible from the “3-D Cluster” tab. New to MIRDcell V3 are views of tomographic sections (illustrated in the worked example below) of the 3-D geometry in the “3D Slice” tab. The tomographic sections of each layer of cells (specified in cell diameters) can be viewed by scrolling the mouse (Fig. 1).

#### SF and Tumor Control Probability (TCP) (New)

In MIRDcell V2.1, the SF of a cell cluster can be plotted as a function of mean activity per cell (Bq), mean absorbed dose to cells (Gy), mean activity per labeled cell (Bq), mean absorbed dose to labeled cells (Gy), and mean absorbed dose to unlabeled cells (Gy). New to MIRDcell V3 are mean decays per cell and mean decays per labeled cell. Also new is presentation of the TCP on the vertical axis, which can be visualized as a function of any of the domains specified above. The TCP is calculated using 2 different approaches. In the first approach, the TCP is calculated using the Poisson model expression (*34*):

$$\text{TCP}\left(D\right)={\left(1-\text{SF}(D)\right)}^{n},$$ Eq. 8

where SF(*D*) is the SF at a mean absorbed dose *D*, and *n* is the number of cells in the cluster. The Poisson model of TCP works under the assumption that the number of surviving cells is Poisson-distributed with an average *n*SF(*D*). The second approach takes the survival probability of each cell into account when calculating the TCP (*22**,**35*). The TCP is calculated using the following expression:

$$\text{TCP}=\overset{n}{\underset{i=1}{\prod }}\left(1-P_{i}\right).$$ Eq. 9

Here, $P_{i}$ is the survival probability of the *i*^th^ cell.

#### Output (New)

Similarly to MIRDcell V2.1, in MIRDcell V3 the output data are written to 2 boxes in the “Output” tab. The right-hand box of the “Output” tab contains the cellular self- and cross-dose S coefficients for all target←source combinations. The left-hand box of the “Output” tab contains most of the information and data used to calculate the absorbed doses and bioeffect. These data are used to create the various plots that are available in the “Multicellular Geometry” tab. New information in the left-hand box of MIRDcell V3 includes additional input information and the option of saving the output data as a .txt file. More granular data are provided, as well absorbed doses from each radiation type, radial dose distributions, and other important data used to make the plots.

## WORKED EXAMPLE

In this section, the overall functionality and accuracy of MIRDcell V3 in predicting biologic response to radiopharmaceuticals is illustrated by a worked example based on data in the literature.

### ^213^Bi Bound to Cells on the Surface of Spherical Cell Clusters

Data published by Kennel et al. (*21*) are used in this example to model the radiotoxicity of ^213^Bi bound to the surface of EMT-6 or LINE-1 tumor cells grown as spheroids. Briefly, monoclonal antibody 13A to murine CD44 was labeled with ^213^Bi (^213^Bi-MAb13A). Only the outer cell layer of the spheroid was labeled, such that the activity was localized to a layer 10 μm from the spheroid surface. The dosimetry was performed using Monte Carlo methods with an assumed nuclear radius of 5.35 μm. The average spheroid diameter in their Figure 6 was 250 μm. On the basis of their Table 4, we estimated that a cluster of this diameter had 3,743 cells. These and other parameters set below were used for both EMT-6 and LINE-1 tumor cells as per Kennel et al. (*21*).

### Methods

**FIGURE 2.**
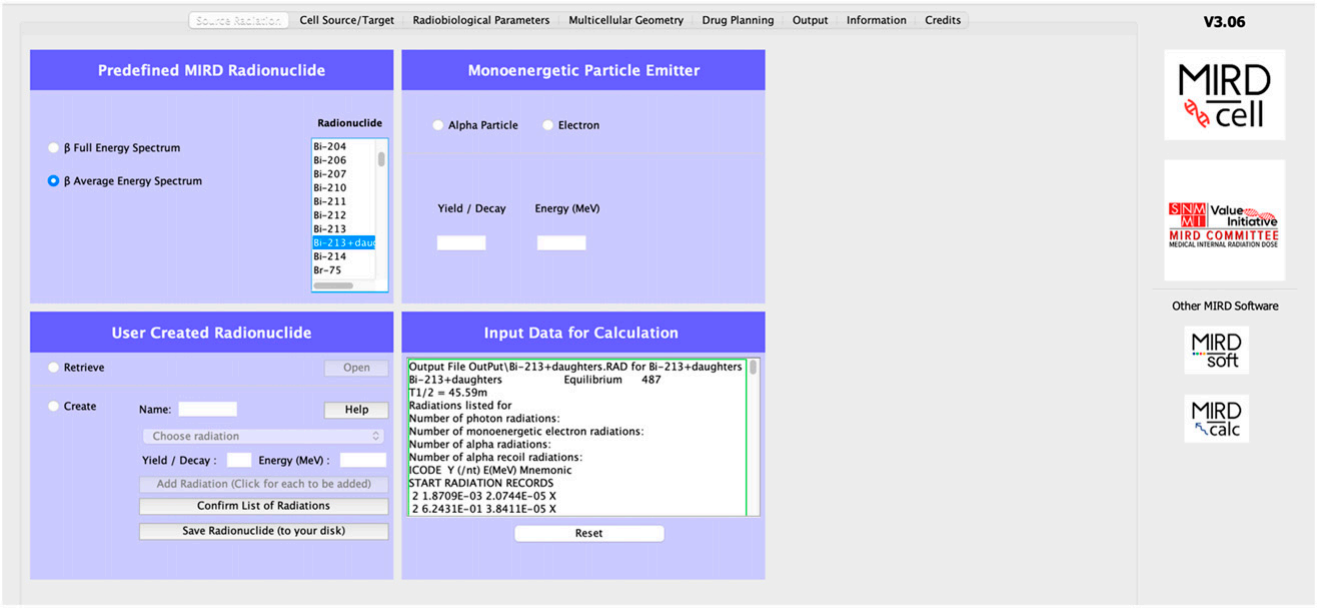
“Source Radiation” tab.

From the “Source Radiation” tab in MIRDcell, the β average-energy spectrum of ^213^Bi plus daughters is selected (Fig. 2). By selecting ^213^Bi plus daughters, all radiation types emitted by the daughters of the ^213^Bi decay chain are considered in the model and the daughters are assumed to be in equilibrium with the parent. The radiation data are displayed in the “Input Data for Calculation” box.

**FIGURE 3.**
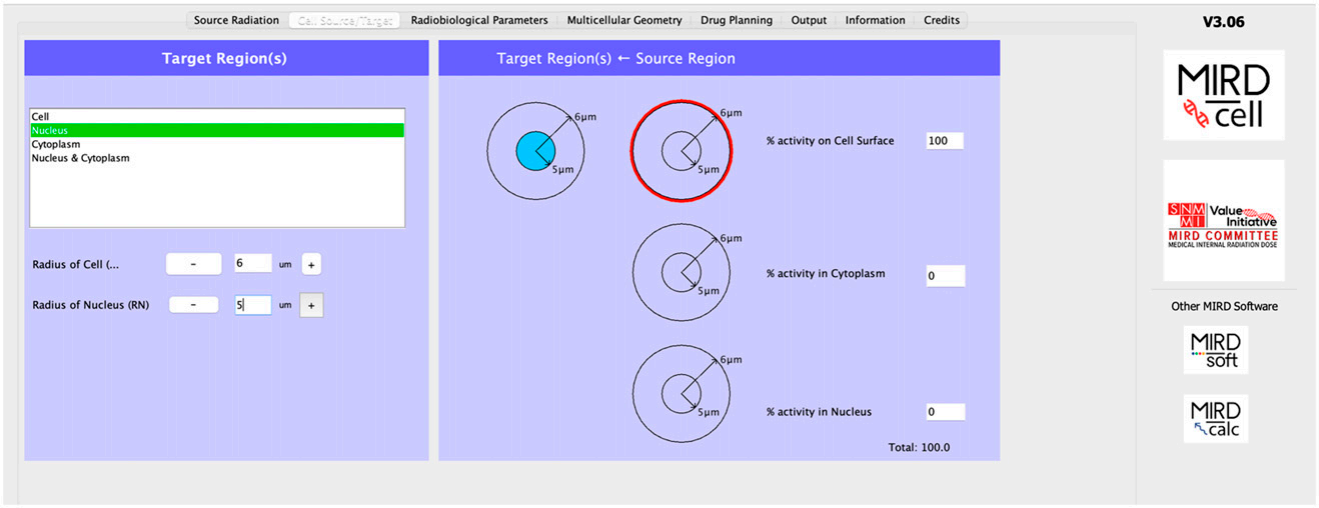
“Cell Source/Target” tab.

In the “Cell Source/Target” tab, the nucleus is selected as the target region and the single source region is the cell surface. The radius of the nucleus is set to 5 μm. The radius of the cell and the distance between cells (μm) in the “Multicellular Geometry” tab are adjusted until the number of cells in the spherical cluster matches the experimental observations (3,473). This requires a cell radius of 6 μm and a distance between cells of 13 μm (Fig. 3).

**FIGURE 4.**
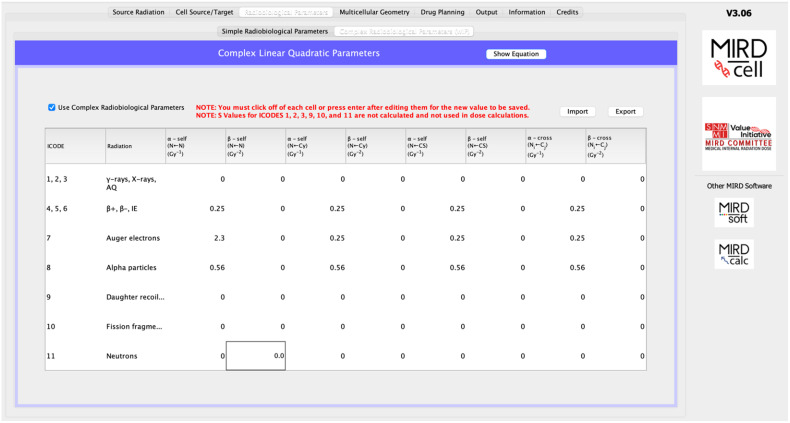
Complex radiobiologic parameters.

Since the ^213^Bi decay chain involves many different radiation types, and the LQ parameters vary depending on the type of radiation and the target←source regions, the “Complex Radiobiologic Parameters” tab is used rather than the “Simple Radiobiologic Parameters” tab (Fig. 4). Kennel et al. (*21*) reported a *D*_0_ of ∼1.8 Gy using a planar α-particle source for both cell lines applied in their experiment. Therefore, the α parameter for α-particles in the LQ model is changed to 1/1.8 Gy^−1^∼ 0.56 Gy^−1^. Default values are kept for the other radiation types. The model was also run with zeros for all the parameters of Auger electrons and β-particles, and the results were the same as when run with default values (i.e., Auger electrons and β-particles play no significant role in the response).

**FIGURE 5.**
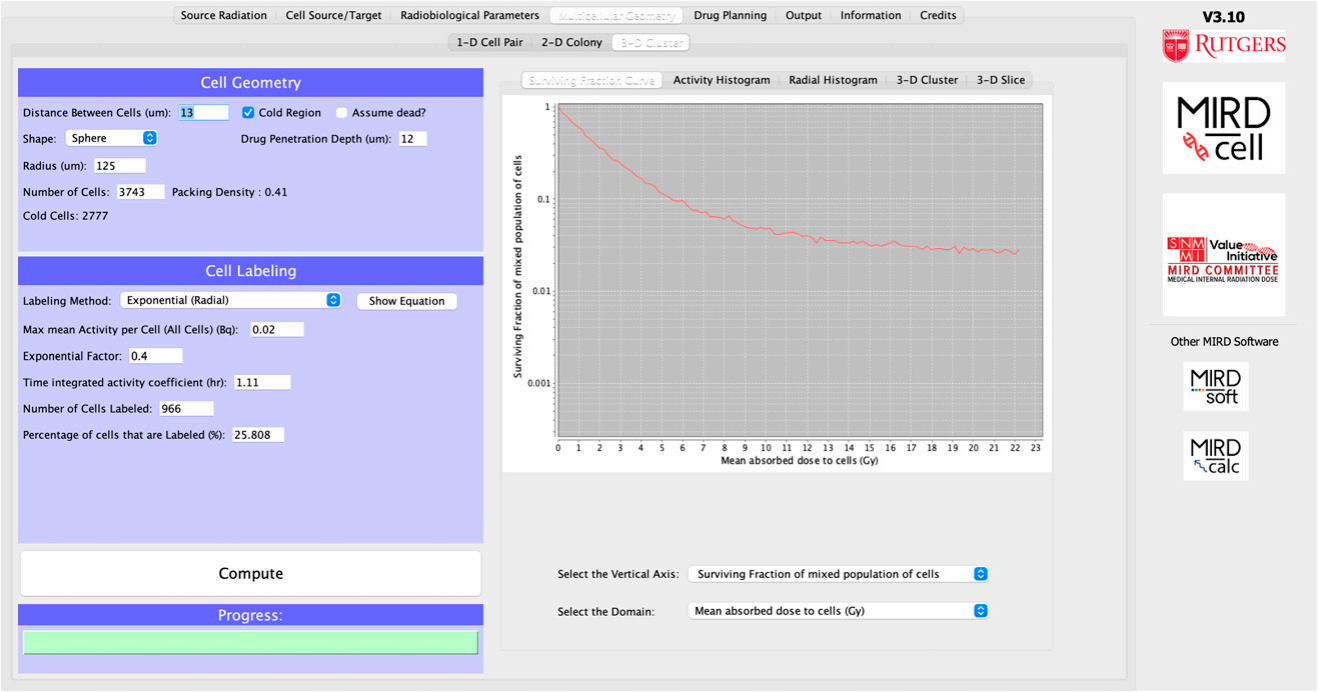
“Multicellular Geometry” tab. SF variation as function of mean activity per cell is shown on right.

**FIGURE 6.**
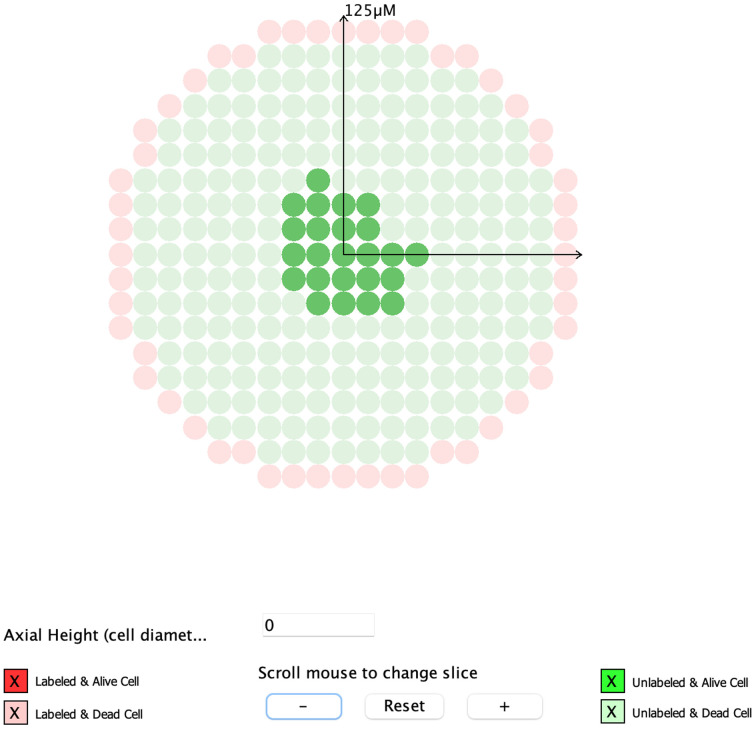
Tomographic section through center of spherical cell cluster illustrating drug penetration depth, labeled cells (red), unlabeled cells (green), alive cells (opaque), and dead cells (translucent). Only unlabeled cells at center of cluster are alive.

From the “Multicellular Geometry” tab, the “3-D Cluster” tab is selected and the radius of the cluster is set to 125 μm. The distance between cells is adjusted until the number of cells matches the experimental observations (described in the “Complex Radiobiological Parameters” section). A drug penetration depth of 12 μm is set, and a radial exponential activity distribution is selected from the drop-down labeling-method menu; the exponential factor is set to 0.4. Since the drug penetrates to only a single cell layer (∼12 μm), the selection of the activity distribution has minimal effect on the rest of the cluster. The time-integrated activity coefficient is set to *T_p_*/ln(2) = 1.11 h (where *T_p_* is the physical half-life of the radionuclide). Even though what ultimately matters is the product of the time-integrated activity coefficient and the maximum mean activity per cell, which provides the mean number of decays per cell (after correcting h to s), it is helpful to know the time-integrated activity coefficient for reasonability checks. The percentage of cells that are labeled in MIRDcell is set to 100%. The maximum mean activity per cell (all cells) (Bq) is adjusted until the maximum mean absorbed dose to cells in the MIRDcell SF curve matches the maximum average dose (Gy) given in Figure 6 of Kennel et al. (*21*). When the “Compute” button is clicked the first time, an error message will pop up indicating that 100% of the cells cannot be labeled because this number exceeds the number of cells within the drug penetration depth; the percentage of labeled cells will be automatically set to the maximum number allowed when the error message is accepted. Therefore, a good rule is to let the program decide the percentage of labeled cells when a drug penetration depth is specified. Alternatively, if there is a specific desired percentage, the value can be set before the “Compute” button is clicked. Once the model is run, the SF as a function of different domains can be visualized under the “SF Curve” tab. The maximum mean activity per cell that matched the desired absorbed dose was 0.02 Bq (Fig. 5). The radial activity histograms and tomographic sections (Fig. 6) of the selected cell cluster geometry are displayed in the “Radial Histogram” and “3-D Slice” tabs, respectively. Tomographic sections of each cell layer can be displayed by specifying the value, in terms of cell diameters, in the box labeled “Axial Height.” Alternatively, the sections can be scrolled through using the wheel of the mouse.

The “Output” tab lists the values of all the parameters used in the model, along with the results. The left panel lists all the output data used for the plots; these data can be viewed under the “Multicellular Geometry” tab. The right panel lists all the self-dose S coefficients and the cross-dose S coefficients as a function of the distance between the center of the source cell and the center of the target cell.

### Results and Comparison with Experimental Observations

**FIGURE 7.**
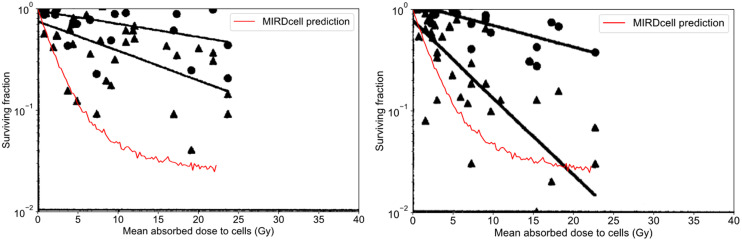
Comparison of MIRDcell prediction with experimental observations. Original plots extracted from Kennel et al. (*21*) have been overlayed with MIRDcell predictions (red). (A) EMT-6 cells. (B) LINE-1 cells. Triangles are data obtained for MAb13A, and circles are those obtained for MAb14, which is nonbinding with tissue. Solid lines are least-squares fits to exponential function provided by Kennel et al. MIRDcell simulation was run for MAb13A cells.

Figure 7 compares the experimental observations for the SF as a function of mean absorbed dose for the 2 cell lines as taken from Figure 6 of Kennel et al. (*21*). The triangles represent the radiolabeled antibody data, and the predictions from MIRDcell are given by the red lines. It can be seen from both plots that the data are better represented by the MIRDcell prediction than by the single-component exponential fit used by Kennel et al. Notably, the MIRDcell prediction for the LINE-1 cells is superior to that for the EMT-6 cells. Of greatest importance to radiopharmaceutical bioeffect modeling is that MIRDcell predicts the appearance of a tail in the curve as the absorbed dose is increased.

Two additional examples are in the supplemental materials. Example 2 predicts the radiotoxicity of ^111^In-epidermal growth factor distributed in spherical cell clusters (Supplemental Figs. 1–7). Example 3 describes the use of several new features in MIRDcell V3 (Supplemental Figs. 8–10). Additional examples, published previously (*2*), are in the user manual.

Also included in the supplemental materials are comparisons between S coefficients calculated for 50-keV electrons with MIRDcell and the Monte Carlo code, TOPAS-nBio (Supplemental Figs. 11–12) (*36*). These S coefficients were used in MIRDcell to generate and compare SF and TCP curves for a 100-μm-radius multicellular cluster with uniform or exponential activity distributions (Supplemental Figs. 13–20). S coefficients were calculated similarly for ^177^Lu and compared (Supplemental Fig. 21). A final comparison for electrons was made with S coefficients that were calculated on the basis of the Emfietzoglou range–energy relationship (Supplemental Fig. 22) (*37*). Lastly, comparisons between S coefficients calculated for 5-MeV α-particles and ^210^Po are made between MIRDcell and TOPAS-nBio (Supplemental Figs. 23–24).

## DISCUSSION

Several other codes for multicellular dosimetry and bioeffect modeling have been published over the years (*5**,**38*–*40*). Charlton published a program for multicellular dosimetry that used analytic approaches to predict cell survival in micrometastases consisting of 2 cell types (*41*). Hobbs et al. created a GEANT4-based program for multicellular dosimetry with features to calculate TCP (*35*). Howell et al. expanded on his earlier work by studying the impact of lognormal distributions of activity among the cell population in multicellular clusters (*42*). Marcatili et al. developed general-purpose software tools to generate randomized 3-D cell culture geometries based on experimentally determined parameters (cell size, cell density, cluster density, average cluster size, cell cumulated activity). Their models were used in conjunction with analytic and Monte Carlo dosimetry calculations to predict the fraction of surviving cells after uptake of ^177^Lu radiopharmaceuticals (*43*). Cai et al. developed a multicellular model that used MCNP radiation transport (*44*). Sizeable differences of up to about 30% in the cross-dose S coefficients produced by their code versus MIRDcell V2.1 were noted. These differences, and their modest impact on SF and TCP, are discussed in the supplemental materials. The most detailed model was published by Raghavan et al. (*45*). The Raghavan model accounts for time-dependent advection and diffusion of radiopharmaceuticals into cells surrounding the cavity that remains after resecting brain tumors. Although MIRDcell does not have a similar capability, we are developing a Python code that processes 3-D activity distribution snapshots over time and calculates the radially dependent time-integrated activity on a cell-by-cell basis.

Although many multicellular dosimetry programs have been developed, they are largely in the hands of their creators and not available widely for general use. Supplemental Table 1 compares the features of MIRDcell with 2 codes that are available for users, namely COOLER (*46*) and PARaDIM (*47*). Although these can accommodate more diverse geometric shapes for the cells, they have a limited scope of other options compared with MIRDcell and they lack user-friendly graphic user interfaces.

Except for the added new features in MIRDcell V3.10, the underlying modeling concepts and assumptions are the same as those of MIRDcell V2.1. The cell and the cell nucleus are still modeled as concentric spheres. The effect of the shape of the cell on the calculated absorbed dose is usually small (*25*), except for certain electron energies that have ranges similar to cellular dimensions (*46*). Furthermore, unlike PARaDIM (*47*), V3 also assumes a constant size for all the cells in the population. Like MIRDcell V2.1, the dosimetry and bioeffect modeling in MIRDcell V3 does not account for the stochastic variations in the number of α-particle decays, hits, and energy deposited. Furthermore, users should be mindful that photons are ignored in V3.10 and earlier versions of MIRDcell; photon contributions to the absorbed dose can become significant for large cluster sizes. MIRDcell V3.10 does not take bystander and abscopal effects into account in the model either (*48*–*50*). Also, any dose rate effects and temporal effects such as proliferation are not explicitly accounted for in modeling the biologic response. However, as mentioned in MIRD pamphlet no. 25 (*2*), this limitation can be compensated for by using suitable values for the LQ parameters in the “Radiobiologic Parameters” tab.

Similarly to MIRDcell V2.1, V3.10 also uses a variation of the LQ model that accounts for self- and cross-doses when modeling the biologic response of cell clusters to different radiation types. The effect of lesion interactions produced by mixtures of self- and cross-dose on biologic response are ignored; rather, their effects are considered independently. New in V3, accommodated by the “Complex Radiobiologic Parameters” tab, is a new target region (cytoplasm) and the capacity to adjust the LQ parameters for each individual radiation type. Again, the effects of each radiation type are treated independently, as are the effects from absorbed doses arising from decays in different source regions. This approach can underestimate the effect, particularly at high doses. However, the exact mechanisms behind interactions with mixed–linear-energy-transfer radiations are not well understood. Various theoretic formulations for bioeffect modeling of mixed radiations have been proposed by both experimental and theoretic groups over the years (*51*–*53*). They include the addition of interaction terms between the various radiation insults that can arise. Although our worked examples here and in the supplemental materials show that the present MIRDcell bioeffect models behave satisfactorily, the introduction of interaction terms is under investigation for possible inclusion in MIRDcell algorithms.

## CONCLUSION

Given the highly nonuniform cellular exposures received in nuclear medicine, designing treatment plans for therapeutic radiopharmaceuticals is challenging. Therefore, the revisions to this software application were developed to improve visualization and understanding of the impact of radionuclide choice, distribution of activity in and among cells, cell dimensions, intercell distances, cluster size, and radiobiologic response parameters on the capacity to kill populations of cells. These parameters can play a substantial role in determining the SF of cells and TCP. Accordingly, MIRDcell is a versatile software tool that can be used for educational purposes and design of RPTs.
